# Vitamin C is associated with improved outcomes in patients with sepsis-induced myocardial injury: insights from the MIMIC-IV database

**DOI:** 10.1186/s12879-025-12377-1

**Published:** 2025-12-24

**Authors:** Rui Gong, Zifeng Huang, Jinyi Zhao, Meng Tang, Fei Mu, Kexin Sun, Chen Cui, Zhen Yan, Jingwen Wang

**Affiliations:** 1 Department of Pharmacy, Xijing Hospital, Fourth Military Medical University, Xi’an, Shaanxi Province 710032 China; 2 School of Pharmacy, Shanxi Medical University, Taiyuan, Shanxi Province 030000 China

**Keywords:** Vitamin C, Sepsis-induced myocardial injury (SIMI), All-cause mortality, ICU, MIMIC-IV database

## Abstract

**Background:**

Vitamin C (VC) is a water-soluble, essential micronutrient with multiple important physiological functions. Numerous studies have shown that VC deficiency is closely associated with adverse clinical outcomes in critically ill patients. Due to the extreme oxidative stress and inflammatory response associated with sepsis and its complications, which lead to a rapid depletion of VC. This study aims to explore whether VC supplementation can reduce the mortality of patients with sepsis-induced myocardial injury (SIMI).

**Methods:**

This study used the MIMIC-IV database to extract data of patients with SIMI and conducted a retrospective cohort study. The patients were divided into the VC administration group (VC group) and the non-VC administration group (non-VC group). The study adopted the multiple imputation method to handle the missing data and balanced the baseline characteristics between the two groups using propensity score matching (PSM). The primary outcome was 28-day all-cause mortality, and the secondary outcome included 90-day and 1-year all-cause mortality. Univariate and multivariable Cox proportional hazards regression models were used to evaluate the association between VC administration and mortality in patients with SIMI, and further analyzed the impact of VC dosage on clinical outcomes. The results were visualized using Kaplan-Meier survival curve. Subgroup analysis was conducted to explore the differences in therapeutic efficacy of VC among different populations.

**Results:**

There were a total of 1488 patients in the final cohort who met the SIMI criteria. After PSM, 129 patients who received VC were matched with 129 non-VC patients. Kaplan-Meier survival curve analysis showed that the VC group had significantly reduced mortality rates at 28 days (27.132% vs. 9.302%, *p* < 0.001), 90 days (33.333% vs. 16.279%, *p* < 0.001) and 1 year (44.186% vs. 27.132%, *p* < 0.001). Multivariable Cox regression analysis further confirmed that VC administration significantly reduced 28-day (HR = 0.20, 95% CI [0.10, 0.41]), 90-day (HR = 0.25, 95% CI [0.14, 0.43]) and 1-year mortality risk (HR = 0.32, 95% CI [0.20, 0.50]). Regarding dosage, the 28-day and 90-day mortality of SIMI patients in the high-dose group were obviously lower than those in the low-dose group, whereas no significant difference was observed in 1-year mortality between the two groups. Subgroup analysis showed a consistent benefit of VC across most populations.

**Conclusion:**

The administration of VC was associated with significantly reduced 28-day, 90-day, and 1-year mortality in patients with SIMI, and high-dose group conferred a greater benefit than low-dose group in the short term.

**Supplementary Information:**

The online version contains supplementary material available at 10.1186/s12879-025-12377-1.

## Introduction

Sepsis is a critical syndrome caused by pathogen infection, which triggers an uncontrolled, dysregulated host immune response. This in turn leads to systemic excessive inflammation, microcirculation disorders and coagulation dysfunction. This pathological cascade reaction can directly result in progressively worsening multiple organ dysfunction syndrome (MODS), with clinical manifestations including life-threatening organ function impairments such as respiratory failure, circulatory shock, and damage to the liver, kidney and heart [1–3]. Among these, sepsis-induced myocardial injury (SIMI) is a serious complication with high mortality in this syndrome [4], with an incidence as high as 40–50% [5, 6]. Although SIMI is often considered a potentially reversible complication in the clinal course of sepsis, its actual prognosis is poor, with an in-hospital mortality of approximately 35% and even as high as 51% within one year [7]. At present, supportive care is still the main treatment for SIMI. Although potential therapies such as positive inotropic drugs (such as dopamine, levosimendan), beta blockers, and anti-inflammatory drugs have been studied and reported [8–10], the uncertainty of their efficacy and the risk of serious adverse events require careful consideration of the risk and benefit in clinical decision-making, highlighting the urgency of in-depth analysis of the pathological mechanisms of SIMI and the development of targeted therapies.

The pathophysiological mechanisms of SIMI involve complex interactions between inflammatory responses and myocardial dysfunction [11]. The classic pathological paradigm of sepsis has long been dominated by the “excessive inflammatory response” theory, which emphasizes that a systemic pro-inflammatory cytokine storm is the core mechanism of organ damage. Based on this, early clinical trials aimed to suppress the host inflammatory response using anti-inflammatory strategies such as glucocorticoids, nonsteroidal anti-inflammatory drugs (NSAIDs), and targeted inflammatory pathway inhibitors, but the results were minimal [12]. With the deepening understanding of the pathophysiology of SIMI, new therapies such as statins [13], dexmedetomidine [14], vitamin C (VC) [15, 16], and thiamine [17] have become the focus of exploration.

In treatment strategies for critically ill patients, naturally water-soluble VC, as an essential micronutrient, demonstrates pivotal potential as an adjunctive therapy [18–21]. However, its role in sepsis/SIMI treatment remains controversial. Previous studies have shown that intravenous administration of high-dose VC in severely septic patients with VC deficiency can dose-dependently improve organ function (reduced SOFA score), accompanied by a decrease in inflammation and endothelial injury markers [22], and even a small trial and subsequent meta-analysis suggested that it can significantly reduce mortality in patients with septic shock and significantly reduce their requirement for and duration of vasopressors use (such as norepinephrine) [23, 24]. These findings indicated that VC administration may become the standard treatment for sepsis. Conversely, multiple studies have shown that VC administration confers no significant benefit in patients with sepsis [2, 25–29], and may even significantly increases the risk of serious adverse events such as hyperoxaluria and acute kidney injury [30]. Existing studies have predominantly focused on the general sepsis population but have paid less attention to SIMI patients-an important subgroup that combines unique pathophysiological mechanisms with higher clinical risk. It is worth noting that studies have found a negative correlation between plasma VC concentrations lower than normal levels and the incidence of multiple organ failure in sepsis patients [31], and some studies suggested that VC may have a protective effect on the heart in sepsis patients [32]. Therefore, it is crucial to elucidate the role of VC in improving the prognosis of SIMI patients, the findings of which may revolutionize the management strategies of SIMI. Based on this, this study used MIMIC-IV database to explore the effect of VC administration on the prognosis of patients with SIMI.

## Methods and materials

### Data sources

This study is a retrospective study using the Medical Information Mart for Intensive Care (MIMIC)-IV database (version 3.0). This dataset contains patient information from 2008 to 2019 who were admitted to the intensive care unit (ICU) at the Beth Israel Deaconess Medical Center (BIDMC) in Boston, Massachusetts, including demographic characteristics, vital signs, laboratory indicators, drug prescriptions, nursing records, surgical records, diagnosis, and survival data. A researcher completed the required online course and obtained database access qualification certification (certification number: 74469213). All data were fully anonymized, so it was not necessary to obtain informed consent for this study.

### Study population

The inclusion criteria for this study were adult (≥ 18 years old) patients diagnosed with sepsis and combined with SIMI within 24 h after admission to the ICU, and their ICU stay was between 24 h and 100 days. To avoid measurement bias caused by duplicate entries, if a patient has multiple admission records, only the initial ICU data from their index admission (that is, the first hospitalization recorded in the database) will be used for analysis. Sepsis was defined using the Sepsis-3.0 criteria [1]. The diagnosis of SIMI is based on the worst cardiac troponin T (cTnT) results recorded on the day of admission to the ICU. The 99th percentile of the reference upper limit for cTnT in this center is 0.01 ng/mL, and SIMI is defined as cTnT > 0.01 ng/mL [4, 33–35]. To exclude other factors that may cause abnormal elevation of cTnT, this study excluded patients with the following conditions: acute coronary syndrome, cardiomyopathy, myocarditis, valve disease, endocarditis, pericarditis, chronic obstructive pulmonary disease, chronic heart failure, previous history of cardiac surgery, cardiac arrest before ICU admission, and history of severe tachyarrhythmia, and patients with a Bleeding Academic Research Consortium (BARC) score ≥ 2 during hospitalization.

### Data collection

In this study, the PostgreSQL (version 14.2) database and SQL (structured query language) were used to extract baseline characteristics of patients, including demographic (gender, age and race), vital signs (heart rate (HR), respiratory rate (RR), systolic blood pressure (SBP)‌, diastolic blood pressure (DBP) and oxygen saturation of hemoglobin (SpO2)), laboratory test results (cTnT, hemoglobin (Hb), platelet (PLT), white blood cell count (WBC), blood urea nitrogen (BUN), creatinine (Cr) and lactate (Lac)), comorbidities (chronic kidney disease (CKD), diabetes mellitus (DM), Liver_disease, malignancy at ICU admission, hypertension and dyslipidemia), critical assessment (sequential organ failure assessment (SOFA) and simplified acute physiology score II‌ (SAPS II)), and the length of ICU stay. Baseline treatment includes invasive mechanical ventilation (IMV), peripherally inserted central catheter (PICC), continuous renal replacement therapy (CRRT), application of vasopressors, angiotensin converting enzyme inhibitors (ACEIs), angiotensin II receptor blockers (ARBs), and beta blockers.

### Exposure and outcomes

The exposure variable in this study was the administration of VC in SIMI patients during ICU admission. A daily dose of 6 g or less of VC is considered a low dose, while a higher amount of VC is regarded as a high dose [36, 37]. The 28-day all-cause mortality was the primary outcome of this study, and the secondary outcomes included 90-day and 1-year all-cause mortality.

### Propensity score matching (PSM) analysis

To evaluate the baseline covariate balance, this study calculated the Standardized mean difference (SMD) and the *p*-value of hypothesis testing for most common variables. The key confounding factors included in the assessment are gender, age, race, SOFA score, SAPS II score, Hb, Lac, SpO2, PICC, Vasopressor and CRRT, among others. Therefore, given the above confounding factors were unevenly distributed between groups, we adjusted for these confounding factors using the PSM method [38], aiming to achieve a balanced distribution of all covariates between the VC administration group (VC group) and the non-VC administration group (non-VC group) within the same scoring level by constructing a propensity score model. We employed an algorithm of 1:1 nearest neighbor matching with a caliper width of 0.05 for this analysis. Finally, the effectiveness of PSM was also evaluated by calculating the SMD of the variables.

### Statistical analysis

In this study, the missing rate of variables was < 10%, and we used multiple imputation method to estimate the missing values. The data in this study are expressed as mean ± standard deviation (SD) or median (interquartile range). For group comparisons, categorical variables were described as percentages and chi-square test was used for comparison. For continuous variables, independent T-test and Wilcoxon rank-sum test were selected based on their distribution characteristics. To explore the nonlinear association between cTnT levels and mortality risk in SIMI patients, we used the Restricted Cubic Splines (RCS) curve for fitting. In survival analysis, the baseline variables were initially screened through univariate Cox regression. Subsequently, the variables with *p* < 0.05 in univariate analysis and applicable to clinical practice were included in multivariate Cox regression model to further evaluate their independent effects. The results were expressed as hazard ratio (HR) and 95% confidence interval (CI). To visualize the cumulative mortality at 28 days, 90 days, and 1 year, Kaplan-Meier survival curves were used. Besides, subgroup analysis was performed using multivariate Cox regression analysis according to relevant clinical characteristics. We used R software (v.4.4.3) for statistical analysis and *P* < 0.05 was considered statistically significant.

## Results

### Baseline characteristics of patients with SIMI

**Fig. 1:**
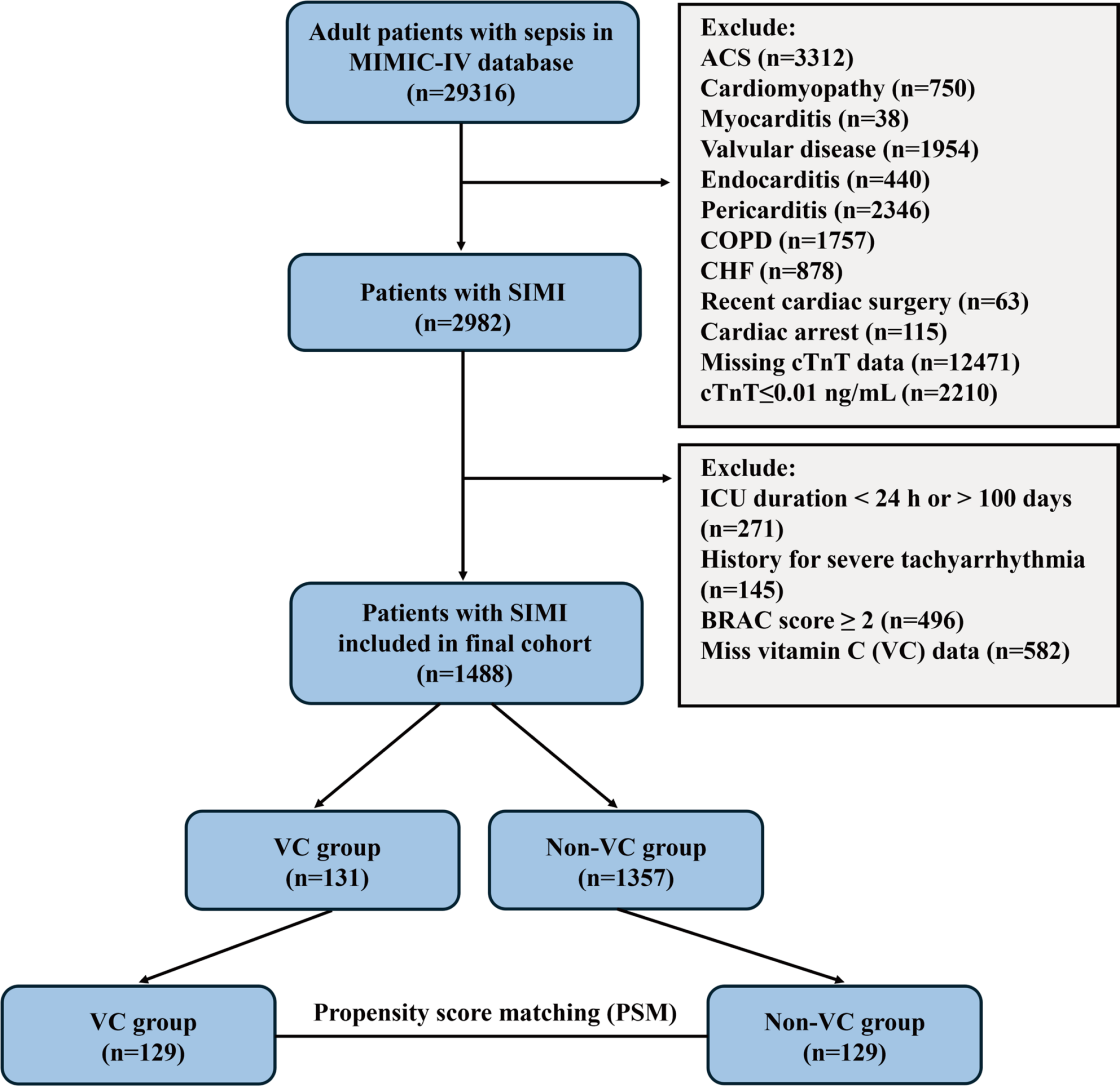
Flow chart of the cohort selection process in this study

This study cohort eventually included 1488 patients who met the diagnostic criteria for SIMI, with the specific screening process shown in Fig. 1. Among them, 131 patients (8.8%) were administered VC during their ICU hospitalization. Before matching, compared to the non-VC group (*n* = 1357), the VC group had significantly lower Hb level (10.500 [8.900, 11.850] vs. 11.400 [9.900, 13.200], *P* < 0.001), HR (86.536 [76.459, 98.774] vs. 88.320 [76.333, 99.706]), and SAPS II score (40.000 [33.000, 54.500] vs. 41.000 [33.000, 52.000]), but higher BUN level (32.000 [17.000, 56.000 vs. 29.000 [19.000, 49.000]) and Cr level (1.700 [1.000, 3.850 vs. 1.500 [1.000, 2.600]). The VC group was also more likely to receive PICC (63 of 131 [48.092%] vs. 455 of 1357 [33.530%]) and CRRT (10 of 131 [7.634%] vs. 31 of 1357 [2.284%]). In terms of comorbidities, the incidence of liver disease (32 of 131 [24.427%] vs. 242 of 1357 [17.833%] and hyperlipidemia (52 of 131 [39.695%] vs. 480 of 1357 [35.372%]) was higher in the VC group than in the non-VC group, while the incidence of hypertension (48 of 131 [36.641%] vs. 577 [42.520%]) was lower. After PSM, a total of 258 patients (129 in each group) were selected for subsequent analysis, and the inter-group biases of most baseline characteristics were effectively balanced. Compared to the matched non-VC group, the VC group still had significantly lower Lac level (1.700 [0.800, 2.500] vs. 2.000 [0.900, 3.700]) and Hb level (10.500 [8.900, 11.800] vs. 11.800 [10.100, 13.200], *P* < 0.001), alongside higher BUN (32.000 [17.000, 56.000] vs. 30.000 [19.000, 54.000]) and Cr level (1.700 [1.000, 3.600] vs. 1.600 [1.000, 2.800]). Moreover, the prevalence of liver disease (32 of 129 [24.806%] vs. 22/129 [17.054%]), hyperlipidemia (52 of 129 [40.310%] vs. 40 of 129 [31.008%]) and CKD (33 of 129 [25.581%] vs. 28 of 129 [21.705%]) increased, while the prevalence of hypertension (47 of 129 [36.434%] vs. 55 of 129 [42.636%]) decreased in the VC group compared with the non-VC group (Table 1).

**Table 1:** Baseline characteristics between the two groups before and after PSM

Characteristic	Original cohort					PSM cohort			
	Non-VC group	VC group	*P*	SMD	Non-VC group		VC group	*P*	SMD
	*N* = 1357	*N* = 131			*N* = 129		*N* = 129		
**Demographic variables**									
Admission age (year), Median (Q1, Q3)	69.159 (58.483, 79.724)	65.747 (56.948, 75.911)	0.034	0.176	69.549 (58.260, 80.827)		65.896 (57.261, 75.929)	0.138	0.154
Gender, N (%)			0.174	0.135				0.256	0.158
Female	597 (43.994%)	49 (37.405%)			59 (45.736%)		49 (37.984%)		
Male	760 (56.006%)	82 (62.595%)			70 (54.264%)		80 (62.016%)		
Race, N (%)			0.091	0.282				0.257	0.289
White	788 (58.069%)	87 (66.412%)			72 (55.814%)		87 (67.442%)		
Black	169 (12.454%)	17 (12.977%)			21 (16.279%)		16 (12.403%)		
Asian	58 (4.274%)	2 (1.527%)			3 (2.326%)		2 (1.550%)		
Hispanic	50 (3.685%)	7 (5.344%)			4 (3.101%)		6 (4.651%)		
Other	292 (21.518%)	18 (13.740%)			29 (22.481%)		18 (13.953%)		
**Vital signs**,** Median (Q1**,** Q3)**									
HR (bpm)	88.320 (76.333, 99.706)	86.536 (76.459, 98.774)	0.410	0.051	86.556 (74.322; 97.769)		86.536 (76.346; 98.308)	0.629	0.060
RR (bpm)	19.593 (17.280, 22.679)	19.852 (16.989, 23.892)	0.554	0.830	19.367 (17.068, 23.071)		19.742 (16.960, 23.568)	0.727	0.053
SBP (mmHg)	113.719 (105.680, 126.678)	112.037 (104.584, 123.939)	0.210	0.104	108.840 (103.368, 123.083)		112.037 (104.625, 123.907)	0.208	0.147
DBP (mmHg)	61.310 (55.375, 68.040)	60.625 (55.707, 68.484)	0.827	0.010	59.875 (53.444, 65.714)		60.569 (55.704, 68.143)	0.211	0.169
SpO2 (%)	97.382 (95.913, 98.600)	96.750 (95.320, 98.226)	0.032	0.062	97.481 (96.042, 98.609)		96.875 (95.385, 98.238)	0.066	0.073
**Comorbidity**,** N (%)**									
CKD			1.000	0.060				0.558	0.091
0	1001 (73.766%)	97 (74.046%)			101 (78.295%)		96 (74.419%)		
1	356 (26.234%)	34 (25.954%)			28 (21.705%)		33 (25.581%)		
Diabetes mellitus			0.113	0.158				1.000	0.017
0	896 (66.028%)	96 (73.282%)			93 (72.093%)		94 (72.868%)		
1	461 (33.972%)	35 (26.718%)			36 (27.907%)		35 (27.132%)		
Liver disease			0.082	0.162				0.168	0.191
0	1115 (82.167%)	99 (75.573%)			107 (82.946%)		97 (75.194%)		
1	242 (17.833%)	32 (24.427%)			22 (17.054%)		32 (24.806%)		
Malignancy			0.253	0.121				0.390	0.128
0	1122 (82.682%)	114 (87.023%)			106 (82.171%)		112 (86.822%)		
1	235 (17.318%)	17 (12.977%)			23 (17.829%)		17 (13.178%)		
Hypertension			0.227	0.120				0.373	0.127
0	780 (57.480%)	83 (63.359%)			74 (57.364%)		82 (63.566%)		
1	577 (42.520%)	48 (36.641%)			55 (42.636%)		47 (36.434%)		
Dyslipidemia			0.373	0.090				0.153	0.194
0	877 (64.628%)	79 (60.305%)			89 (68.992%)		77 (59.690%)		
1	480 (35.372%)	52 (39.695%)			40 (31.008%)		52 (40.310%)		
**Laboratory tests**,** Median (Q1**,** Q3)**									
cTnT (ng/mL)	0.080 (0.040, 0.200)	0.080 (0.045, 0.190)	0.662	0.614	0.080 (0.030, 0.2000		0.080 (0.040, 0.190)	0.502	0.6225
Hb (g/dL)	11.400 (9.900, 13.200)	10.500 (8.900, 11.850)	< 0.001	0.453	11.800 (10.100, 13.200)		10.500 (8.900, 11.800)	< 0.001	0.507
PLT (10^3^/µL)	206.000 (139.000, 280.000)	218.000 (155.000, 308.000)	0.165	0.09	204.000 (131.000, 286.000)		218.000 (153.000, 313.000)	0.170	0.125
WBC (10^3^/µL)	14.100 (9.900, 20.100)	14.500 (10.800, 20.900)	0.330	0.100	14.800 (10.400, 21.800)		14.500 (10.800, 21.100)	0.828	0.097
BUN (mg/dL)	29.000 (19.000, 49.000)	32.000 (17.000, 56.000)	0.497	0.115	30.000 (19.000, 54.000)		32.000 (17.000, 56.000)	0.708	0.075
Cr (mg/dL)	1.500 (1.000, 2.600)	1.700 (1.000, 3.850)	0.169	0.200	1.600 (1.000, 2.800)		1.700 (1.000, 3.600)	0.435	0.231
Lac (mmol/L)	1.800 (0.000, 3.600)	1.700 (0.800, 2.500)	0.464	0.068	2.000 (0.900, 3.700)		1.700 (0.800, 2.500)	0.218	0.054
**Critical assessment**,** Median (Q1**,** Q3)**									
SOFA	7.000 (5.000, 10.000)	8.000 (4.000, 13.000)	0.094	0.233	7.000 (5.000, 11.000)		7.000 (4.000, 13.000)	0.820	0.022
SAPS II	41.000 (33.000, 52.000)	40.000 (33.000, 54.500)	0.810	0.080	44.000 (35.000, 53.000)		39.000 (33.000, 54.000)	0.189	0.125
**Treatments**,** N (%)**									
IMV			0.775	0.035				0.526	0.095
0	593 (43.699%)	55 (41.985%)			49 (37.984%)		55 (42.636%)		
1	764 (56.301%)	76 (58.015%)			80 (62.016%)		74 (57.364%)		
PICC			0.001	0.300				0.166	0.188
0	902 (66.470%)	68 (51.908%)			80 (62.016%)		68 (52.713%)		
1	455 (33.530%)	63 (48.092%)			49 (37.984%)		61 (47.287%)		
CRRT			0.002	0.250				0.036	0.298
0	1326 (97.716%)	121 (92.366%)			128 (99.225%)		121 (93.798%)		
1	31 (2.284%)	10 (7.634%)			1 (0.775%)		8 (6.202%)		
Vasopressors			0.141	0.140				0.277	0.154
0	1177 (86.735%)	107 (81.679%)			99 (76.744%)		107 (82.946%)		
1	180 (13.265%)	24 (18.321%)			30 (23.256%)		22 (17.054%)		
**Medication**,** N (%)**									
ACEIs			0.553	0.067				1.000	< 0.010
0	1127 (83.051%)	112 (85.496%)			110 (85.271%)		110 (85.271%)		
1	230 (16.949%)	19 (14.504%)			19 (14.729%)		19 (14.729%)		
ARBs			0.433	0.100				1.000	0.048
0	1289 (94.989%)	127 (96.947%)			126 (97.674%)		125 (96.899%)		
1	68 (5.011%)	4 (3.053%)			3 (2.326%)		4 (3.101%)		
beta blockers			0.418	0.082				0.803	0.046
0	597 (43.994%)	63 (48.092%)			59 (45.736%)		62 (48.062%)		
1	760 (56.006%)	68 (51.908%)			70 (54.264%)		67 (51.938%)		

Abbreviations: PSM, propensity score matching; HR, heart rate; RR, respiratory rate; SBP, systolic blood pressure; DBP, diastolic blood pressure; SpO2, oxygen saturation of hemoglobin; Cr, creatinine; BUN, blood urea nitrogen; WBC, white blood cell; PLT, platelet; Hb, hemoglobin; Lac, lactate; CKD, chronic kidney disease; DM, diabetes mellitus; IMV, invasive mechanical ventilation; CRRT, continuous renal replacement therapy; PICC, peripherally inserted central catheter; SOFA, sequential organ failure assessment; SAPS II, simplified acute physiology score II‌; ACEIs, angiotensin converting enzyme inhibitors; ARBs, angiotensin II receptor blockers; SMD, standardized mean differences

### Association between VC administration and mortality in patients with SIMI

**Fig. 2:**
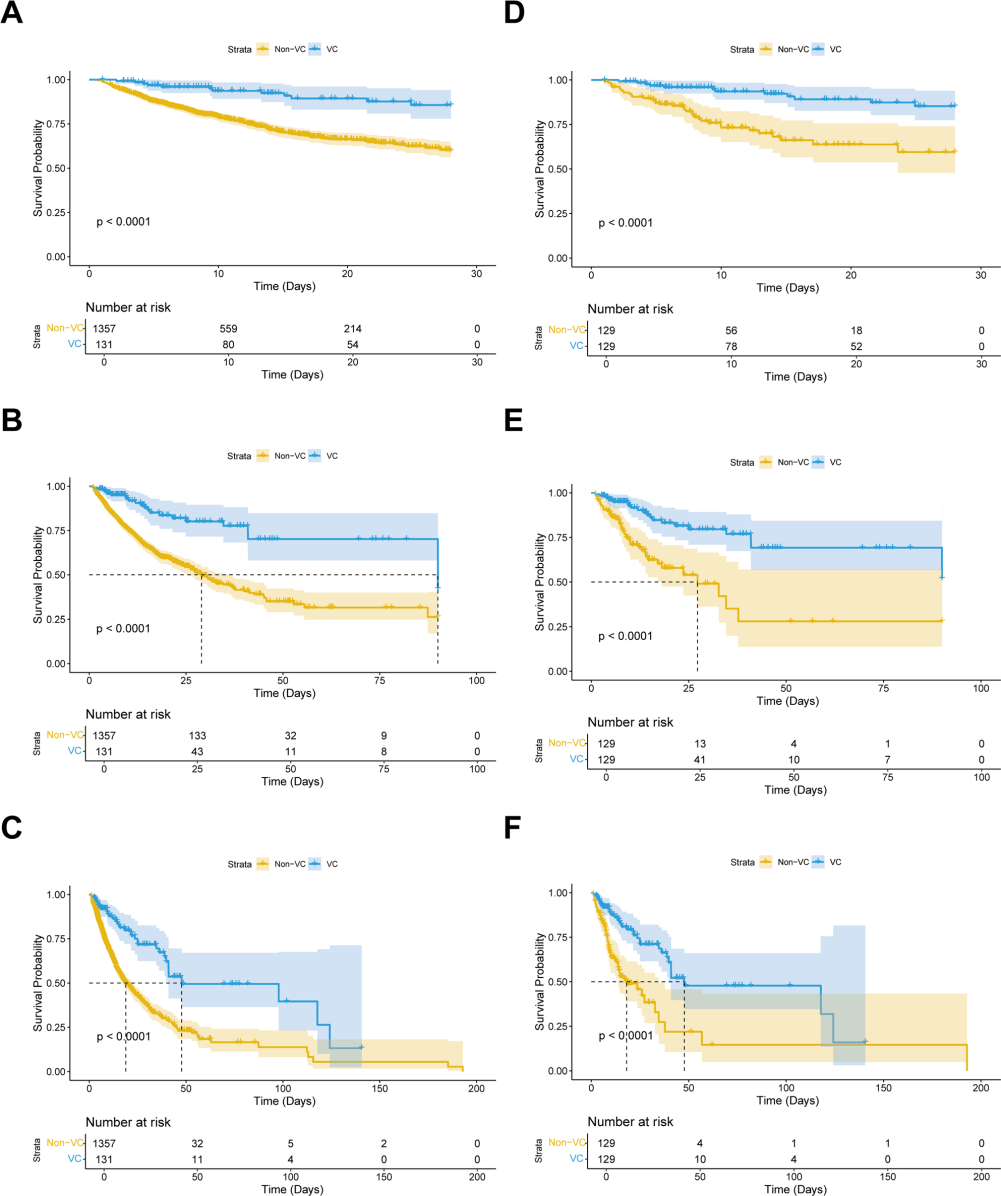
Kaplan-Meier survival curves of the non-VC group and VC group. (**A**) 28-day mortality before PSM; (**B**) 90-day mortality before PSM; (**C**) 1-year mortality before PSM; (**D**) 28-day mortality after PSM; (**E**) 90-day mortality after PSM; (**F**) 1-year mortality after PSM. Abbreviations: VC, vitamin C

Next, we compared the 28-day, 90-day, and 1-year mortality between the VC and non-VC groups. In the original cohort, the 28-day, 90-day, and 1-year mortality in the VC group were 9.160% (12/131), 16.794% (22/131), and 27.481% (36/131), respectively, while those in the non-VC group were 23.950% (325/1357) (*P* < 0.001), 30.582% (415/1357) (*P* = 0.001), and 40.678% (552/1357) (*P* = 0.004), indicating a significant reduction in mortality in the VC group at 28 days, 90 days, and 1 year. After PSM, this association persisted, with the VC group showing lower mortality at 28 days (9.302% vs. 27.132%, *P* < 0.001), 90 days (16.279% vs. 33.333%, *P* < 0.001), and 1 year (27.132% vs. 44.186%, *P* < 0.001) (Table 2). Figure 2 displays the Kaplan-Meier curve for 28-day, 90-day, and 1-year mortality stratified by VC administration in the original or matched cohort. These results suggested a stable association between VC administration and reduced mortality in patients with SIMI.

**Table 2:** Clinical outcomes of SIMI before or after PSM

Mortality	Original cohort			PSM cohort		
	Non-VC group	VC group	*P* value	Non-VC group	VC group	*P* value
28-day mortality	325 (23.950%)	12 (9.160%)	< 0.001	35 (27.132%)	12 (9.302%)	< 0.001
90-day mortality	415 (30.582%)	22 (16.794%)	0.001	43 (33.333%)	21 (16.279%)	< 0.001
1-year mortality	552 (40.678%)	36 (27.481%)	0.004	57 (44.186%)	35 (27.132%)	< 0.001

Abbreviations: SIMI, sepsis-induced myocardial injury; PSM, propensity score matching; VC, vitamin C

**Fig. 3:**
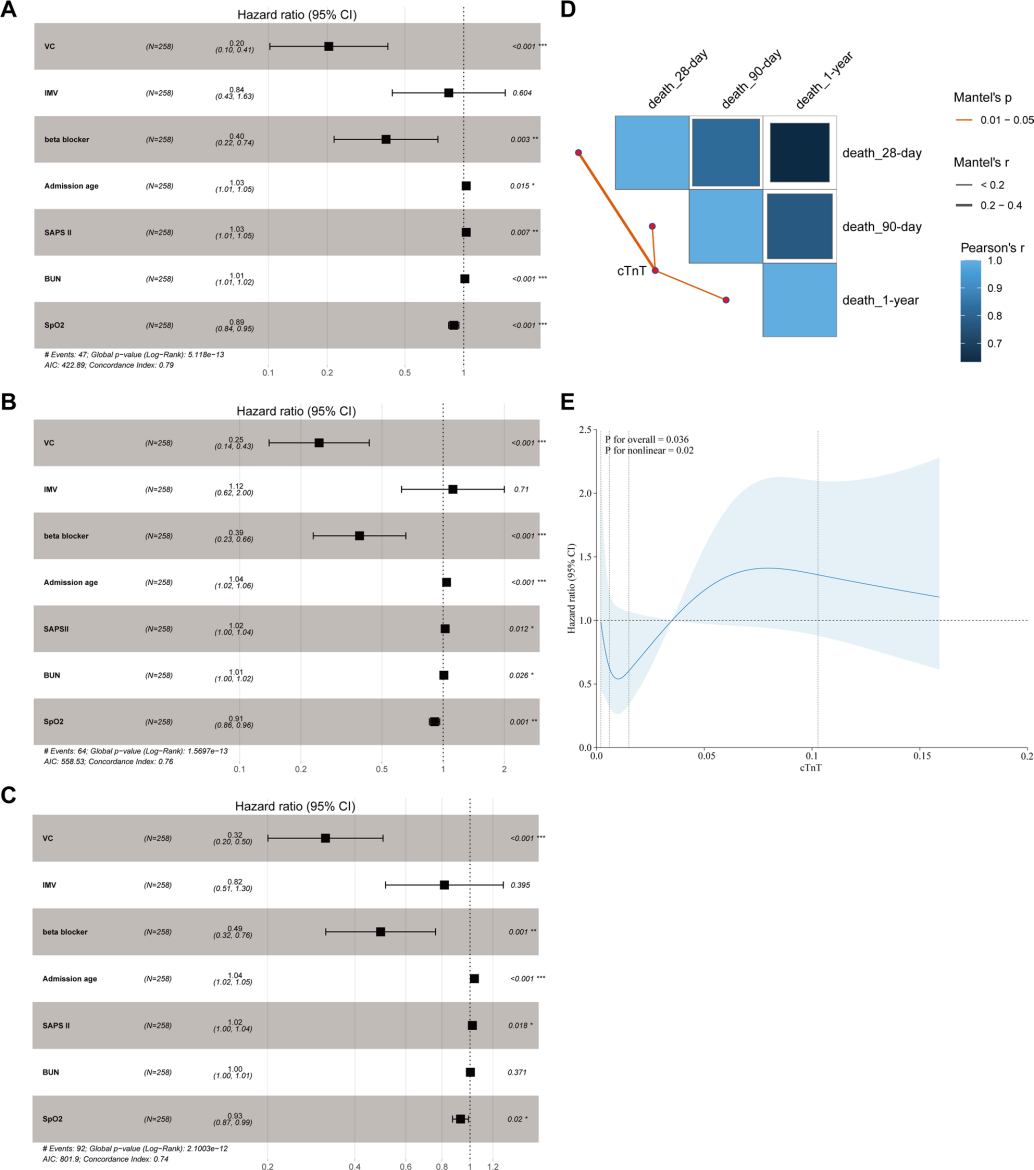
VC administration or cTnT level was significantly correlated with mortality in SIMI patients. (**A**) The multivariate Cox proportional hazards model analysis of the effect of VC administration on 28-day mortality after PSM; (**B**) The multivariate Cox proportional hazards model analysis of the effect of VC administration on 90-day mortality after PSM; (**C**) The multivariate Cox proportional hazards model analysis of the effect of VC administration on 1-year mortality after PSM. (**D**) Butterfly plot of correlation between cTnT levels and mortality (28-day, 90-day, and 1-year) in SIMI patients. (**E**) The RCS curve of the association between cTnT levels and mortality in SIMI patients. The variables “IMV”, “beta blocker”, “Admission age”, “SAPS II”, “BUN” and “SpO2” were the covariates, and cTnT was the main variable, and the optimal number of nodes was 3 according to AIC value

The univariate Cox regression analysis identified several factors associated with 28-day, 90-day, and 1-year mortality before and after PSM (Supplementary Table 1). Afterwards, to adjust for potential confounding, we built multivariate Cox proportional hazards models that included VC administration and known clinically relevant covariates, such as age, SAPS II score, BUN, SpO2, as well as the use of IMV and beta blockers, etc. The forward stepwise selection method was adopted to screen the variables, with minimizing the Akaike Information Criterion (AIC) value as the optimization objective, and the proportional hazards (PH) assumption analysis was carried out. Ultimately, we identified VC administration, IMV, beta blocker, admission age, SAPS II, BUN levels and SpO2 levels as the independent influencing factors for 28-day, 90-day, and 1-year mortality in SIMI patients. We plotted a time-dependent receiver operating characteristic (timeROC) curve to illustrate the changes in model prediction accuracy over time. The results showed that the area under the curve (AUC) of the model at 28 days, 90 days, and 1 year were 0.887, 0.811, and 0.739, respectively (Figure S1A). To visualize the model, we integrated a risk nomogram of independent predictive factors associated with mortality in SIMI patients (Figure S1B), and the calibration curve revealed that the predicted survival was highly consistent with the actual observed values (Figure S1C), indicating that the constructed prediction model had favorable discriminatory ability for predicting mortality risk (especially short-term) in SIMI patients. We further analyzed and found that VC administration significantly reduced all-cause mortality at 28 days, 90 days, and 1 year in both the original cohort and the matched cohort of SIMI patients. The specific HR were as follows: the HR for 28-day mortality was 0.22 (95% CI [0.13, 0.40], *P* < 0.001) in the original cohort and 0.20 (95% CI [0.10, 0.41], *P* < 0.001) in the matched cohort; The HR of 90-day mortality were 0.27 (95% CI [0.18, 0.42], *P* < 0.001) and 0.25 (95% CI [0.14, 0.43], *P* < 0.001), respectively; and the HR of 1-year mortality were 0.35 (95% CI [0.24, 0.49], *P* < 0.001) and 0.32 (95% CI [0.20, 0.50], *P* < 0.001), respectively (Fig. 3A-C and Figure S2). Finally, we assessed the relationship between baseline cTnT levels and mortality. Higher cTnT levels were significantly associated with increased mortality risk at all timepoints (Fig. 3D and Supplementary Table 2). The RCS curve analysis revealed a non-linear relationship between cTnT level and 28-day mortality. Specifically, when the baseline cTnT level exceeded 0.035 ng/mL, the mortality of patients significantly increased (Fig. 3E).

### Association of VC dose with mortality

**Fig. 4:**
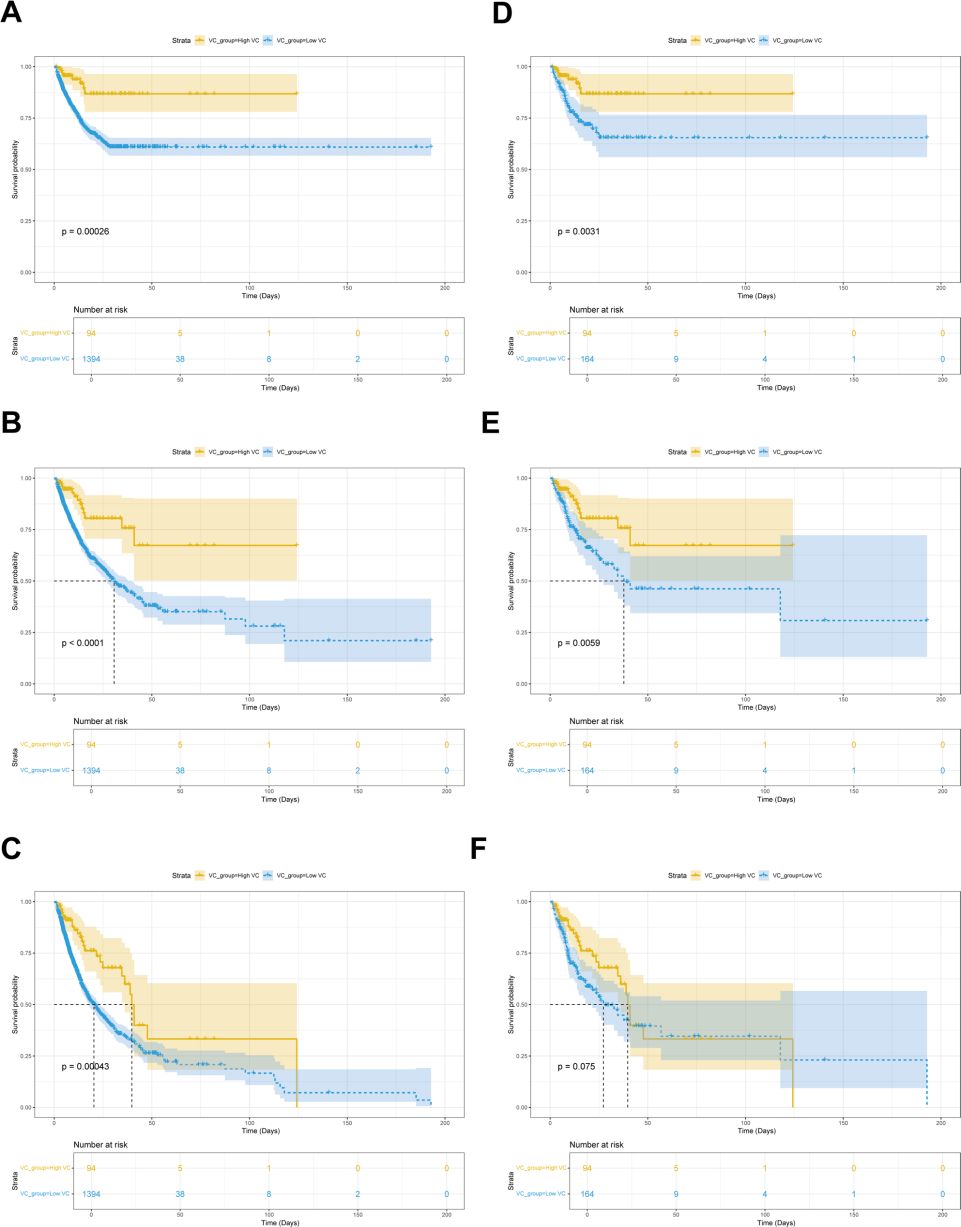
Kaplan-Meier survival curves of 28-day, 90-day, and 1-year mortality in SIMI patients with low-dose and high-dose VC administration. (**A**) 28-day mortality before PSM; (**B**) 90-day mortality before PSM; (**C**) 1-year mortality before PSM; (**D**) 28-day mortality after PSM; (**E**) 90-day mortality after PSM; (**F**) 1-year mortality after PSM

To further explore the protective effect of VC dosage on patients with SIMI, the patients were divided into low-dose (VC ≤ 6 g per day) and high-dose (VC > 6 g per day) [36, 39], and found that the patients in the high-dose VC group had obviously lower Hb level (10.450 (9.000, 11.900) vs. 11.400 (9.900, 13.100); *P* < 0.001) and Lac level (1.450 (0.150, 2.250) vs. 1.800 (0.000, 3.600); *P* = 0.013), and SAPS II score (37.000 (31.000, 48.250) vs. 42.000 (33.000, 52.000); *P* = 0.011) compared with the low-dose VC group. The 28-day mortality (8.511% vs. 23.601%, *P* = 0.001), 90-day mortality (14.894% vs. 30.344%, *P* = 0.002), and 1-year mortality (28.723% vs. 40.244%, *P* = 0.036) were significantly lower in the high-dose group than in the low-dose group. However, after PSM between the low-dose and high-dose groups, the difference in 28-day and 90-day mortality persisted (28-day: 8.511% vs. 23.780%, *P* = 0.004; 90-day: 14.894% vs. 30.488%, *P* = 0.008), but the difference in 1-year mortality was no longer significant (28.723% vs. 39.634%, *P* = 0.104) (Supplementary Table 3). Kaplan-Meier survival curves indicated that in the original cohort, the 28-day, 90-day and 1-year survival rates of patients in the high-dose group were significantly better than those in the low-dose group. Similarly, in the PSM cohort, there was a significant difference in 28-day and 90-day mortality between the two groups, but no significant difference in 1-year mortality (*P* = 0.075) (Fig. 4). Notably, the association between higher VC doses and lower short-term mortality observed in the treatment population deserves further investigation.

### Subgroup analysis

**Fig. 5:**
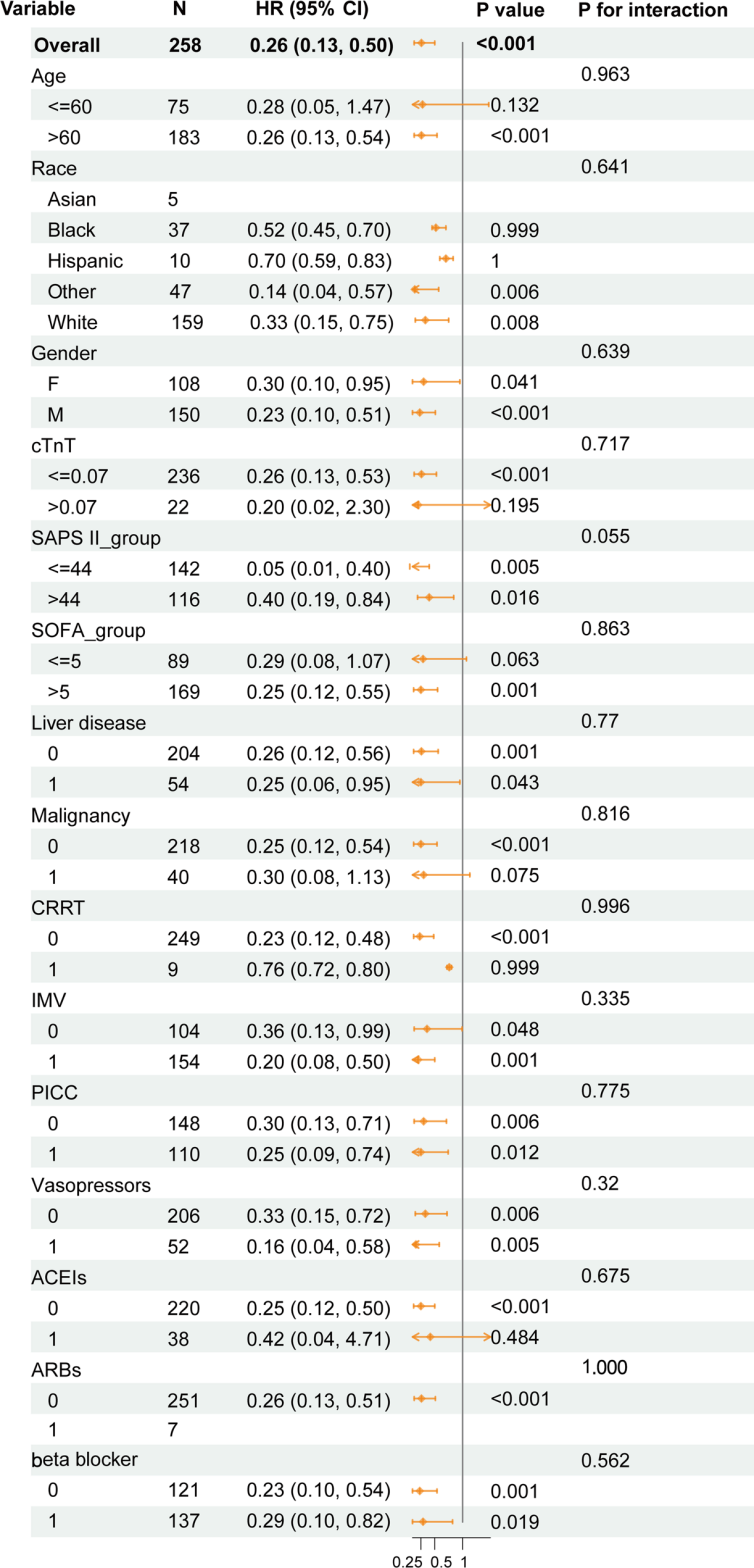
Subgroup analysis of the relationship between VC administration and 28-day mortality in SIMI patients. Abbreviations: SIMI, sepsis-induced myocardial injury; cTnT, cardiac Troponin T; SOFA, Sequential Organ Failure Assessment; SAPS, Simplified Acute Physiology Score; CRRT, continuous renal replacement therapy; IMV, invasive mechanical ventilation; PICC, peripherally inserted central catheter; ACEIs, angiotensin-converting enzyme inhibitors; ARBs, angiotensin II receptor blockers

We conducted subgroup analysis to explore the potential changes in the association between VC administration and 28-day mortality in different clinical subgroups of patients with SIMI. The result was shown in Fig. 5. The overall analysis in the matched cohort (*N* = 258) indicated that VC administration was associated with a significantly lower risk of 28-day mortality of SIMI patients (HR = 0.26, 95% CI [0.13, 0.50]; *P* < 0.001). We then stratified the population by age, race, gender, cTnT level, SAPS II score, SOFA score, and several comorbidities and treatments. Crucially, no significant interactions were observed in all the pre-specified subgroups (all *P* for interaction > 0.05), suggesting that the observed association between VC administration and reduced 28-day mortality was consistent across these different patient characteristics. Although the HR value of the SAPS II ≤ 44 subgroup was lower, the interaction test did not reach statistical significance (*P* = 0.055 for interaction), indicating that this numerical difference may be accidental. Similarly, regardless of baseline cTnT levels, this association is consistent (*P* = 0.717 for interaction). Taken together, the subgroup analysis found no robust evidence to suggest that the association between VC administration and reduced 28-day mortality differed significantly across the pre-specified patient subgroups. The findings regarding consistency of effect should be considered hypothesis-generating and require validation in future studies.

## Discussion

This large retrospective cohort study, using the MIMIC-IV (version 3.0) database, observed a association between VC administration and reduced all-cause mortality at 28 days, 90 days and 1 year in patients with SIMI. After PSM, this association remained strong, suggesting that VC may play an important role in this high-risk population. Furthermore, exploratory analysis indicated that a higher daily dose of VC might be associated with a reduction in short-term mortality compared to lower dose.

Our findings are biologically plausible and consistent with the evolving understanding of the pathophysiology of SIMI. Although dysregulated inflammatory response (“cytokine storm”) is a key feature, the failure of targeted anti-inflammatory therapy has shifted the focus to severe metabolic and bioenergy depletion within myocardial cells, which are key drivers of systolic dysfunction [12, 40–42]. The multifaceted pharmacological characteristics of VC make it a potential therapeutic agent in this context. Specifically, the mortality reduction observed in our study, particularly with higher doses, could be explained by the pleiotropic effect of VC, not just its antioxidant activity [17, 24, 43]. Moreover, VC maintained vascular tension and microcirculation perfusion by supporting the synthesis of endogenous catecholamines and vasopressin [19, 44–47], thereby protecting organ function. More importantly, as an important cofactor of α-ketoglutarate dependent dioxygenase, VC was involved in epigenetic regulation, hypoxia response, and carnitine synthesis [48–50], which are crucial for restarting cellular metabolism during bioenergetic crises. The high prevalence of VC deficiency in sepsis patients was itself associated with poor prognosis [31, 39, 51–53], providing theoretical basis for supplementation. Our findings suggested that this supplementation may not only correct deficiencies; It can also break the vicious cycle of organ dysfunction by reconstructing cell functions at the epigenetic and metabolic levels, enhancing the energy production of cardiomyocytes and the function of immune cells.

Our study’s findings contribute to a complex and sometimes contradictory body of evidence. They align with a recent single-center randomized controlled trial (RCT) [54]. Other studies have reported that patients with severe sepsis treated with VC had a rapid reduction in SOFA score, significantly reduced proinflammatory biomarkers, such as C-reactive protein and procalcitonin, and reduced vascular endothelial damage [22]. Additionally, supplementing with VC during ICU stay has been shown to be associated with a reduced short-term mortality risk in patients with sepsis associated acute kidney injury [55]. Notably, a recent meta-analysis of RCTs found that specific dosing regimens of intravenous VC were associated with a significant reduction in short-term mortality in sepsis (OR = 0.80, 95% CI [0.65, 0.97]; *p* = 0.03) [56]. Although the references are not entirely consistent, the collective evidence from these clinical and meta-analysis studies supports the potential protective role of VC in sepsis and its related organ damage, including SIMI. However, our findings contrast with several large RCTs and subsequent meta-analyses that failed to demonstrate a mortality benefit for VC in broad sepsis populations [28, 57–59]. In particular, a large-scale phase 3 trial found that the VC group had a higher incidence of death or organ failure at 28 days, but this result may be influenced by baseline imbalances-the treatment group had more severe conditions at enrollment, manifested as higher lac levels, a higher proportion of shock and receiving IMV [30], which likely masked the actual efficacy and even led to a conclusion biased towards being harmful. Furthermore, the therapeutic effect of VC is likely time-sensitive [60] and most pronounced in patients with specific clinical phenotypes, such as SIMI, or those with profound VC deficiency-subgroups often not pre-specified in large trials [28, 39]. Sepsis is highly heterogeneous [58, 61]. Different clinical phenotypes and genetic backgrounds and metabolic differences among individuals may all affect the therapeutic response to VC. Our study, by focusing on SIMI and exploring dose-response, provides a crucial explanatory dimension for these inconsistent trial results. However, unmeasured confounding factors (such as baseline VC level) may affect the results, and prospective trials are needed in the future to verify this dose-response relationship.

Subgroup analysis did not show statistically significant interactions, indicating that the association between VC administration and reduced 28-day mortality was generally consistent among the pre-specified patient subgroups. However, there are several numerical trends worth proposing hypotheses for future research. The estimation of HR value indicated that the VC administration may be more beneficial in elderly patients, which may reflect their higher baseline oxidative stress levels and reduced physiological reserves [62–64]. The associated attenuation observed in patients receiving CRRT has a certain physiological basis, as VC is removed by renal replacement therapy [65, 66], indicating that patients receiving CRRT may require higher doses or continuous infusion of VC to compensate for the loss. The RCS curve revealed a non-linear relationship between cTnT level and mortality. Although VC did not show statistical significance in patients with high cTnT (> 0.07 ng/mL), the trend of risk reduction is worthy of attention. This suggests that high cTnT level represent extreme injury states that may require earlier or more aggressive multimodal intervention.

This study has several limitations. First, although this study controlled for widely known confounding factors through PSM and multiple Cox regression (with subsequent model adjustment for variables that remained different after PSM, such as Lac and Hb), and the main results were robust, the retrospective observational design still cannot completely eliminate the influence of residual confounding and unmeasured potential confounding variables. Notably, we also conducted a multivariate Cox regression analysis directly in the original, unmatched cohort. The results showed that even at an unbalanced baseline, the HR direction, magnitude and statistical significance of VC administration were consistent with those of the 1:1 matched cohort (Fig. 3A-C and Figure S2). Nonetheless, the retrospective design itself is still unable to establish a causal relationship between VC administration and prognosis in patients with SIMI, so the conclusions drawn should be interpreted as a correlation between the two. Prospective RCTs are needed to further validate the impact of VC administration on the clinical outcomes of SIMI patients on the basis of strict control of baseline indicators, unified diagnostic criteria and dosage regimen, and to clarify the best treatment strategy and benefit population. Moreover, VC administration decision may be influenced by unrecorded clinical factors, leading to an overestimation of efficacy associations. Second, although the dichotomy of VC dose in this study provides strong evidence for the initial correlation, it fails to describe the continuous dose-response relationship in detail, and the determination of the exact optimal treatment dose remains to be solved in future prospective studies. Third, there is currently no international standard for the diagnosis of SIMI. This study only uses cTnT > 0.01 ng/mL as the population definition basis, which may introduce diagnostic bias and limit the generalization of results. Fourth, although we ensure that myocardial injury can be attributed to sepsis through strict exclusion criteria, completely excluding all potential confounding factors (especially diseases lacking specific coding such as stress cardiomyopathy) in retrospective designs remains a challenge. Fifth, the widespread absence of baseline levels of VC may affect the accuracy of analysis results and the strength of biological explanations for the protective effects of VC. Additionally, the database only provides in-hospital mortality status and does not include specific causes of death and survival data after discharge, which limits the evaluation of long-term efficacy and cause specific outcomes of VC. Meanwhile, the subgroup analysis results may be subject to statistical uncertainty due to sample size limitations, and the lack of continuity of treatment information after discharge also hinders in-depth analysis of long-term outcomes.

In summary, this study provides strong observational evidence for the association between VC administration and improved survival rates in patients with SIMI. These findings challenge the comprehensive negative conclusions of some RCTs by emphasizing the importance of patient selection and medication administration. We strongly recommend that prospective RCTs be initiated to finally confirm the efficacy of VC in SIMI patients. This is not only an important supplement to the current limitations, but also a key step to promote clinical transformation. Future RCTs should focus on determining the optimal administration strategy (including dosage and duration of administration), and include the clinical outcomes including mortality (such as in-hospital mortality, 6-month survival rate after discharge and improvement of cardiac function classification) as the evaluation criteria. Meanwhile, the diagnostic criteria (such as the combination of cTnT, BNP and echocardiography) should be strictly controlled, and the baseline level and dynamic data of VC should be collected to accurately identify the subgroup population that is most likely to benefit. Besides, causal inference methods such as mendelian randomization can be used to further explore the potential causal relationship between VC and SIMI prognosis in the future, so as to provide more reliable evidence support for the clinical application of VC in the treatment of SIMI.

## Conclusion

In summary, this large retrospective study demonstrated that VC administration was significantly associated with reduced 28-day, 90-day, and 1-year mortality in patients with SIMI. Exploratory analysis further indicated that there was a potential association between higher daily doses and improved short-term outcomes compared to lower doses. These findings provide supporting evidence for the current debate on VC in sepsis and emphasize that SIMI is a patient subgroup that may require targeted investigation. Given the high safety and multiple potential mechanisms of action of VC, this study lays a theoretical foundation for conducting large-scale, multi-center RCTs in the future. Future research should focus on clarifying the causal relationship between VC administration and clinical outcomes, determining its optimal administration strategy, and identifying the patient phenotypes most likely to benefit, thereby promoting the precise application of VC in the treatment of SIMI.

## Supplementary Information

Below is the link to the electronic supplementary material.

Supplementary Material 1

## Data Availability

All relevant data for this study are included in the main manuscript or supplementary information files, and are available upon request from the corresponding author.

## Abbreviations

ACEIs: Angiotensin converting enzyme inhibitors
ARBs: Angiotensin II receptor blockers
AIC: Akaike Information Criterion
AUC: Area Under the Curve
BIDMC: Beth Israel Deaconess Medical Center
BUN: Blood urea nitrogen
cTnT: Cardiac troponin T
Cr: Creatinine
CKD: Chronic kidney disease
CRRT: Continuous renal replacement therapy
CI: Confidence interval
DBP: Diastolic blood pressure
DM: Diabetes mellitus
HR: Heart rate
Hb: Hemoglobin
HR: Hazard ratio
IMV: Invasive mechanical ventilation
ICU: Intensive care unit
IQR: Interquartile range
Lac: Lactate
MODS: Multiple organ dysfunction syndrome
MIMIC-IV: Medical Information Mart for Intensive Care-IV
NSAIDs: Nonsteroidal anti-inflammatory drugs
PLT: Platelet
PICC: Peripherally inserted central catheter
PSM: Propensity score matching
PSM: Propensity score matching
RBC: Red blood cell count
RR: Respiratory rate
RCS: Restricted Cubic Splines
RCT: Randomized controlled trial
SOFA: Sequential Organ Failure Assessment
SAPS II: Simplified Acute Physiology Score II
SBP: Systolic blood pressure
SIMI: Sepsis-induced myocardial injury
SpO2: Oxygen saturation of hemoglobin
SD: Standard deviation
SMD: Standardized mean difference
timeROC: Time-dependent receiver operating characteristic
VC: Vitamin C
WBC: White blood cell count
