# Supplementary material for: Vitamin C is associated with improved outcomes in patients with sepsis-induced myocardial injury: insights from the MIMIC-IV database
Source: BMC Infect Dis. 2025 Dec 24;26:171. doi: 10.1186/s12879-025-12377-1 (PMC12849488; doi:10.1186/s12879-025-12377-1)
Supplement: Supplementary file 1 — Supplementary Material 1 [file 12879_2025_12377_MOESM1_ESM.docx]

**Vitamin C is associated with improved outcomes in patients with sepsis-induced myocardial injury: insights from the MIMIC-IV database**

Rui Gong^a#^, Zifeng Huang^a,b#^, Jinyi Zhao^a#^, Meng Tang^a^, Fei Mu^a^, Kexin Sun^a^, Chen Cui^a^, Zhen Yan^a^, Jingwen Wang^a*^

^a^ Department of Pharmacy, Xijing Hospital, Fourth Military Medical University, Xi'an, Shaanxi Province, 710032, China

^b^ School of Pharmacy, Shanxi Medical University, Shanxi Province, 030000, China

# Contributed equally.

* Corresponding author: Jingwen Wang, Department of Pharmacy, Xijing Hospital, Fourth Military Medical University, Xi'an, China. Email: wangjingwen8021@163.com.

**Supplementary Figures**

**
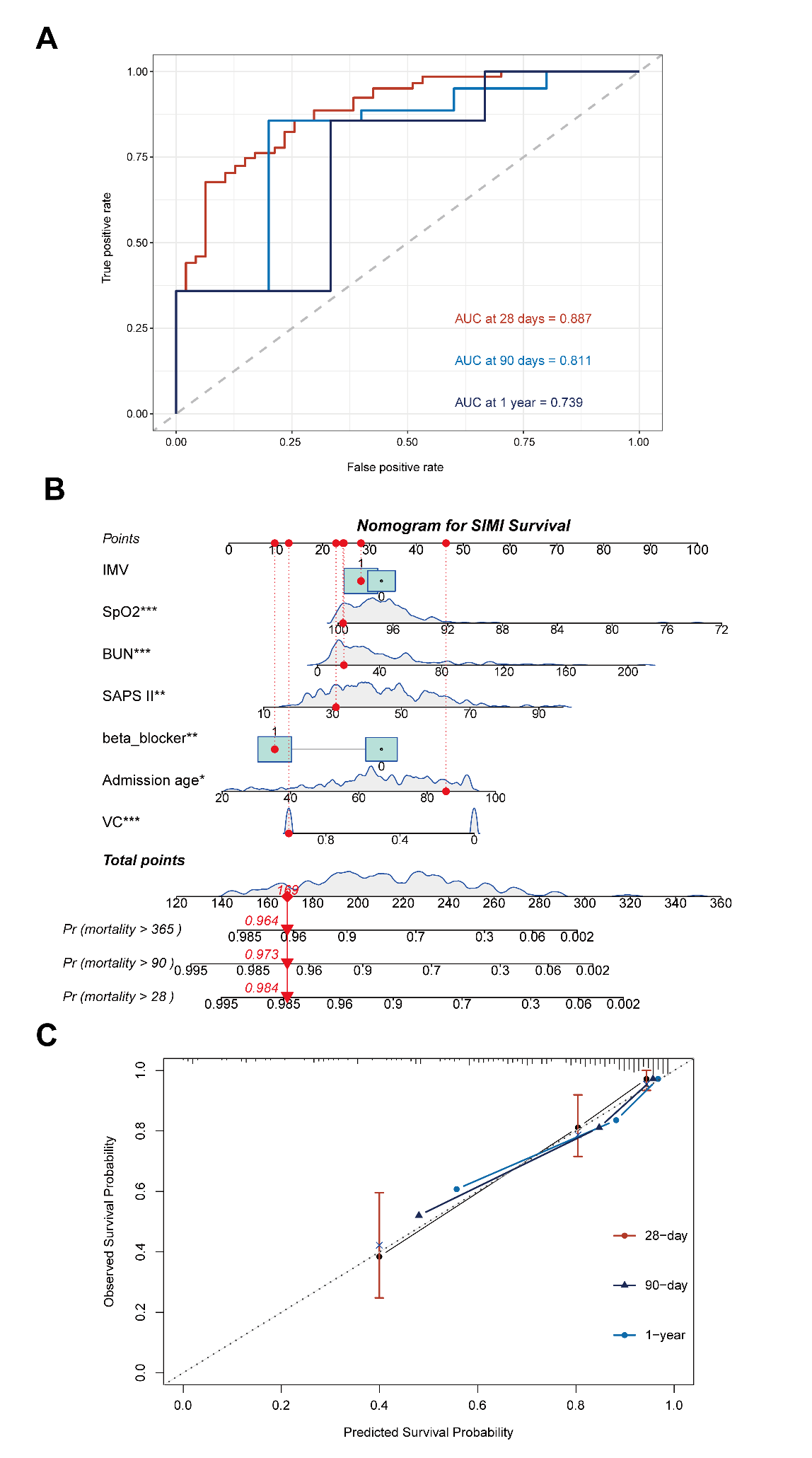
**

**Supplementary Figure 1 Comprehensive Models analysis for predicting the probability of mortality in SIMI patients.** (**A**) The time-dependent ROC curves of the multivariate regression model for predicting the survival of SIMI patients, and the ROC curves for predicting the 28-day (AUC = 0.887), 90-day (AUC = 0.811) and 1-year (AUC = 0.739) survival rates, respectively. (**B**) The nomogram model for predicting 28-day, 90-day and 1-year mortality of patients with SIMI. When using it, drawing a vertical line to the point axis from each risk factor and scoring the corresponding points. After the points of each variable were added to obtain a total score, draw a vertical line again to the total point axis to correspond the probability of 28-day, 90-day and 1-year mortality. (**C**) The calibration curve of the nomogram predicted and actually measured the survival probabilities of SIMI patients at 28 days, 90 days, and 1 year after PSM. IMV, invasive mechanical ventilation; SpO2, saturation of peripheral oxygen; BUN, blood urea nitrogen; SAPS II, simplified acute physiology score II‌; VC, vitamin C; AUC, area under the ROC curve.


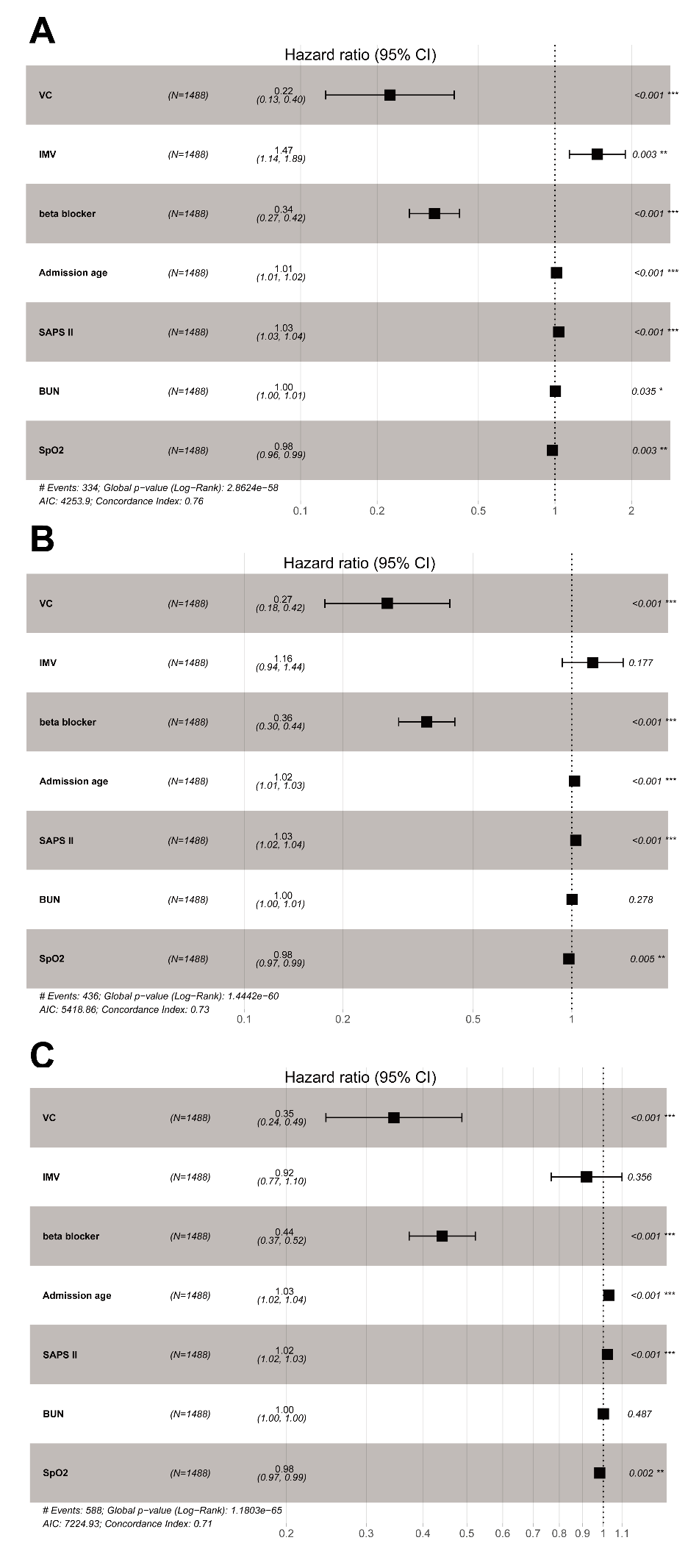


**Supplementary Figure 2 The Multivariate Cox proportional hazards model analysis of the effect of VC administration on mortality in SIMI patients before PSM.** (**A**). 28-day mortality. (**B**) 90-day mortality. (**C**) 1-year mortality. IMV, invasive mechanical ventilation; SpO2, saturation of peripheral oxygen; BUN, blood urea nitrogen; SAPS II, simplified acute physiology score II‌; VC, vitamin C; HR, hazard ratio; CI, confidence interval.

**Supplementary Tables**

**Supplementary Table 1 Univariable Cox regression in before and after PSM cohorts.**

| Variables | Original cohort | | | | | | PSM cohort | | | | | |
| --- | --- | --- | --- | --- | --- | --- | --- | --- | --- | --- | --- | --- |
|  | 28-day mortality | | 90-day mortality | | 1-year mortality | | 28-day mortality | | 90-day mortality | | 1-year mortality | |
|  | HR (95% CI) | *P* value | HR (95% CI) | *P* value | HR (95% CI) | *P* value | HR (95% CI) | *P* value | HR (95% CI) | *P* value | HR (95% CI) | *P* value |
| **Demographic variables** |  |  |  |  |  |  |  |  |  |  |  |  |
| Age, years | 1.020 (1.010, 1.030) | <0.001 | 1.030 (1.020, 1.030) | <0.001 | 1.030 (1.030, 1.040) | <0.001 | 1.030 (1.010, 1.050) | <0.01 | 1.040 (1.020, 1.050) | <0.001 | 1.040 (1.020, 1.050) | <0.001 |
| Gender | 0.953 (0.768, 1.180) | 0.659 | 1.020 (0.844, 1.230) | 0.836 | 1.010 (0.861, 1.190) | 0.867 | 1.520 (0.828, 2.770) | 0.177 | 1.300 (0.782, 2.160) | 0.313 | 1.190 (0.780, 1.810) | 0.423 |
| Race |  |  |  |  |  |  |  |  |  |  |  |  |
| Black | 0.423 (0.217, 0.822) | 0.011 | 0.499 (0.280, 0.889) | 0.018 | 0.570 (0.347, 0.935) | 0.026 | - | - | - | - | - | - |
| Hispanic | 0.639 (0.277, 1.480) | 0.295 | 0.781 (0.385, 1.580) | 0.493 | 0.767 (0.412, 1.430) | 0.405 | - | - | - | - | - | - |
| Other | 1.500 (0.862, 2.610) | 0.151 | 1.310 (0.793, 2.180) | 0.29 | 1.110 (0.708, 1.730) | 0.656 | - | - | - | - | - | - |
| White | 0.763 (0.442, 1.320) | 0.331 | 0.813 (0.497, 1.330) | 0.41 | 0.881 (0.572, 1.360) | 0.566 | - | - | - | - | - | - |
| **Vital signs** |  |  |  |  |  |  |  |  |  |  |  |  |
| HR (bpm) | 1.01 (1.00-1.02) | <0.01 | 1.000 (0.999, 1.010) | 0.094 | 1.000 (0.996, 1.010) | 0.867 | 1.000 (0.985, 1.020) | 0.810 | 1.000 (0.987, 1.020) | 0.902 | 0.992 (0.980, 1.000) | 0.188 |
| RR (bpm) | 1.06 (1.03-1.08) | <0.001 | 1.040 (1.020, 1.060) | <0.001 | 1.020 (1.010, 1.040) | <0.01 | 1.070 (1.010, 1.130) | 0.021 | 1.06 (1.010, 1.110) | 0.015 | 1.020 (0.977, 1.060) | 0.387 |
| SpO2 (%) | 0.990 (0.976, 1.000) | 0.183 | 0.991 (0.978, 1.000) | 0.177 | 0.990 (0.979, 1.000) | 0.065 | 0.864 (0.807, 0.925) | <0.001 | 0.884 (0.825, 0.948) | <0.001 | 0.920 (0.855, 0.990) | 0.026 |
| SBP (mmHg) | 0.986 (0.980, 0.991) | <0.001 | 0.988 (0.983, 0.993) | <0.001 | 0.993 (0.988, 0.997) | <0.01 | 0.971 (0.949, 0.994) | 0.013 | 0.982 (0.964, 1.000) | 0.049 | 0.991 (0.976, 1.010) | 0.211 |
| DBP (mmHg) | 0.985 (0.975, 0.995) | <0.01 | 0.985 (0.976, 0.993) | <0.001 | 0.984 (0.977, 0.992) | <0.001 | 0.955 (0.925, 0.986) | <0.01 | 0.956 (0.929, 0.983) | <0.01 | 0.967 (0.946, 0.990) | <0.01 |
| Temperature (℃) | 0.969 (0.958, 0.981 | <0.001 | 0.977 (0.966, 0.989) | <0.001 | 0.983 (0.972, 0.994) | <0.01 | 0.975 (0.931, 1.020) | 0.272 | 0.980 (0.935, 1.030) | 0.411 | 0.988 (0.943, 1.030) | 0.596 |
| **Laboratory tests** |  |  |  |  |  |  |  |  |  |  |  |  |
| Cr (mg/dL) | 0.984 (0.941, 1.030) | 0.479 | 0.965 (0.926, 1.010) | 0.092 | 0.976 (0.943, 1.010) | 0.159 | 0.987 (0.877, 1.110) | 0.822 | 0.929 (0.826, 1.040) | 0.218 | 0.930 (0.843, 1.030) | 0.151 |
| BUN (mg/dL) | 1.01 (1.01, 1.01) | <0.001 | 1.010 (1.000, 1.010) | <0.001 | 1.000 (1.000, 1.010) | <0.001 | 1.010 (1.010, 1.020) | <0.001 | 1.010 (1.000, 1.010) | 0.031 | 1.000 (0.997, 1.010) | 0.400 |
| WBC (10^3^/μL) | 1.000 (0.999, 1.010) | 0.131 | 1.000 (0.996, 1.010) | 0.742 | 0.999 (0.994, 1.000) | 0.530 | 1.000 (0.990, 1.010) | 0.779 | 0.999 (0.987, 1.010) | 0.811 | 0.996 (0.986, 1.010) | 0.509 |
| PLT (10^3^/μL) | 0.999 (0.998, 1.000) | 0.109 | 0.999 (0.999, 1.000) | 0.116 | 1.000 (0.999, 1.000) | 0.225 | 0.999 (0.997, 1.000) | 0.443 | 1.000 (0.999, 1.000) | 0.440 | 1.000 (0.999, 1.000) | 0.498 |
| Hb (g/dL) | 1.040 (0.995, 1.090) | 0.083 | 1.030 (0.989, 1.070) | 0.159 | 1.010 (0.981, 1.050) | 0.413 | 1.100 (0.974, 1.250) | 0.122 | 1.070 (0.961, 1.190) | 0.220 | 1.030 (0.939, 1.120) | 0.563 |
| Lac (mmol/L) | 1.04 (1.03, 1.05) | <0.001 | 1.030 (1.020, 1.040) | <0.001 | 1.020 (1.010, 1.040) | 0<0.001 | 1.070 (0.993, 1.160) | 0.076 | 1.030 (0.956, 1.110) | 0.430 | 0.988 (0.921, 1.060) | 0.743 |
| **Comorbidities** |  |  |  |  |  |  |  |  |  |  |  |  |
| CKD | 0.641 (0.489, 0.841) | <0.01 | 0.729 (0.580, 0.915) | <0.01 | 0.862 (0.714, 1.040) | 0.120 | 0.848 (0.422, 1.710) | 0.644 | 0.956 (0.536, 1.710) | 0.879 | 0.990 (0.612, 1.600) | 0.967 |
| Liver disease | 1.260 (0.981, 1.630) | 0.070 | 1.200 (0.961, 1.500) | 0.108 | 0.977 (0.798, 1.200) | 0.821 | 0.936 (0.465, 1.880) | 0.852 | 0.799 (0.433, 1.480) | 0.474 | 0.782 (0.468, 1.310) | 0.349 |
| DM | 0.776 (0.611, 0.986) | 0.038 | 0.891 (0.726, 1.090) | 0.270 | 1.010 (0.850, 1.200) | 0.913 | 0.598 (0.289, 1.240) | 0.166 | 0.766 (0.429, 1.370) | 0.366 | 0.782 (0.482, 1.270) | 0.317 |
| Hypertension | 1.010 (0.815, 1.260) | 0.909 | 0.989 (0.816, 1.200) | 0.909 | 1.010 (0.859, 1.200) | 0.879 | 0.691 (0.374, 1.280) | 0.237 | 0.823 (0.494, 1.370) | 0.455 | 0.764 (0.494, 1.180) | 0.226 |
| Dyslipidemia | 0.900 (0.716, 1.130) | 0.369 | 0.930 (0.761, 1.140) | 0.482 | 1.020 (0.857, 1.210) | 0.841 | 0.595 (0.303, 1.170) | 0.132 | 0.695 (0.399，1.210) | 0.198 | 0.736 (0.464, 1.170) | 0.193 |
| Malignancy | 1.580 (1.230, 2.020) | <0.001 | 1.440 (1.150, 1.790) | <0.01 | 1.580 (1.310, 1.900) | <0.001 | 1.770 (0.916, 3.400) | 0.090 | 1.300 (0.705, 2.390) | 0.404 | 1.390 (0.845, 2.290) | 0.195 |
| **Critical assessment** |  |  |  |  |  |  |  |  |  |  |  |  |
| SAPS II | 1.04 (1.04, 1.05) | <0.001 | 1.040 (1.030, 1.040) | <0.001 | 1.030 (1.020, 1.030) | <0.001 | 1.040 (1.020, 1.050) | <0.001 | 1.030 (1.020, 1.050) | <0.001 | 1.020 (1.010, 1.040) | <0.001 |
| SOFA | 1.120 (1.100, 1.150) | <0.001 | 1.080 (1.050, 1.100) | <0.001 | 1.030 (1.010, 1.050) | <0.01 | 1.060 (0.997, 1.120) | 0.063 | 1.030 (0.977, 1.080) | 0.294 | 0.978 (0.936, 1.020) | 0.325 |
| **Treatments** |  |  |  |  |  |  |  |  |  |  |  |  |
| IMV | 1.420 (1.120, 1.800) | <0.01 | 1.060 (0.871, 1.300) | 0.545 | 0.800 (0.677, 0.946) | <0.01 | 0.894 (0.490, 1.630) | 0.715 | 0.994 (0.581, 1.700) | 0.981 | 0.698 (0.454, 1.070) | 0.101 |
| CRRT | 1.880 (1.180, 2.990) | <0.01 | 1.340 (0.864, 2.060) | 0.193 | 0.916 (0.599, 1.400) | 0.686 | 0.822 (0.199, 3.400) | 0.787 | 0.760 (0.237, 2.440) | 0.644 | 0.480 (0.150, 1.530) | 0.216 |
| Vasopressor | 2.880 (2.280, 3.620) | <0.001 | 2.020 (1.630, 2.500) | <0.001 | 1.450 (1.190, 1.770) | <0.001 | 1.870 (1.030, 3.400) | 0.041 | 1.420 (0.836, 2.400) | 0.196 | 0.900 (0.554, 1.460) | 0.672 |
| PICC | 0.891 (0.713, 1.110) | 0.308 | 0.870 (0.716, 1.060) | 0.159 | 0.758 (0.640, 0.898) | <0.01 | 0.506 (0.275, 0.931) | 0.028 | 0.600 (0.361, 0.995) | 0.048 | 0.636 (0.417, 0.971) | 0.036 |
| **Medication** |  |  |  |  |  |  |  |  |  |  |  |  |
| ACEIs | 0.208 (0.124, 0.349 | <0.001 | 0.311 (0.214, 0.454) | <0.001 | 0.461 (0.350, 0.608) | <0.001 | 0.400 (0.124, 1.290) | 0.125 | 0.646 (0.279, 1.500) | 0.310 | 0.809 (0.418, 1.560) | 0.528 |
| ARBs | 0.177 (0.057, 0.551) | <0.01 | 0.271 (0.121, 0.606) | <0.01 | 0.582 (0.359, 0.943) | 0.028 | - | - | 0.874 (0.213, 3.590) | 0.851 | 0.589 (0.145, 2.400) | 0.461 |
| beta blocker | 0.367 (0.294, 0.459) | <0.001 | 0.409 (0.338, 0.495) | <0.001 | 0.507 (0.430, 0.597) | <0.001 | 0.515 (0.287, 0.922) | 0.026 | 0.572 (0.349, 0.937) | 0.027 | 0.686 (0.453, 1.040) | 0.074 |

**Abbreviations:** PSM, propensity score matching; HR, heart rate; RR, respiratory rate; SBP, systolic blood pressure; DBP, diastolic blood pressure; SpO2, oxygen saturation of hemoglobin; Cr, creatinine; BUN, blood urea nitrogen; WBC, white blood cell; PLT, platelet; Hb, hemoglobin; Lac, lactate; CKD, chronic kidney disease; DM, diabetes mellitus; IMV, invasive mechanical ventilation; CRRT, continuous renal replacement therapy; PICC, peripherally inserted central catheter; SOFA, sequential organ failure assessment; SAPS II, simplified acute physiology score II‌; ACEIs, angiotensin converting enzyme inhibitors; ARBs, angiotensin II receptor blockers; HR, hazard ratio.

**Supplementary Table 2** The correlation between cTnT level and mortality of patients with SIMI.

| Variables | Outcome | r | *P* value | r range | *P* range |
| --- | --- | --- | --- | --- | --- |
| cTnT | 28-day mortality | 0.247 | 0.0171 | 0.2-0.4 | 0.01-0.05 |
|  | 90-day mortality | 0.146 | 0.024 | < 0.2 | 0.01-0.05 |
|  | 1-year mortality | 0.0823 | 0.044 | < 0.2 | 0.01-0.05 |

**Supplementary Table 3 Baseline characteristics of the VC groups (by dose) before and after PSM.**

| Variables | Original cohort | | | | PSM cohort | | | |
| --- | --- | --- | --- | --- | --- | --- | --- | --- |
|  | Low does-VC group  (N = 1394) | High dose-VC group  (N = 94) | *P* value | SMD | Low does-aspirin group  (N = 164) | High dose-aspirin group  (N = 94) | *P* value | SMD |
| **Demographic variables** |  |  |  |  |  |  |  |  |
| Age, years, Median (Q1, Q3) | 68.873 (58.355, 79.578) | 66.406 (57.503, 78.781) | 0.426 | 0.352 | 67.244 (57.818, 79.016) | 66.406 (57.503, 78.781) | 0.856 | 0.253 |
| Gender, N (%) |  |  | 0.354 | 0.73 |  |  |  |  |
| Male | 784 (56.241%) | 58 (61.702%) |  |  | 92 (56.098%) | 58 (61.702%) | 0.455 | 0.693 |
| Female | 610 (43.759%) | 36 (38.298%) |  |  | 72 (43.902%) | 36 (38.298%) |  |  |
| Race, N (%) |  |  | 0.066 | 0.325 |  |  | 0.145 | 0.909 |
| White | 814 (58.250%) | 63 (67.021%) |  |  | 96 (58.537%) | 63 (67.021%) |  |  |
| Black | 172 (12.339%) | 14 (14.894%) |  |  | 23 (14.024%) | 14 (14.894%) |  |  |
| Asian | 58 (4.161%) | 2 (2.128%) |  |  | 3 (1.829%) | 2 (2.128%) |  |  |
| Hispanic | 52 (3.730%) | 5 (5.319%) |  |  | 5 (3.049%) | 5 (5.319%) |  |  |
| Others | 300 (21.521%) | 10 (10.638%) |  |  | 37 (22.561%) | 10 (10.638%) |  |  |
| **Vital signs, Median (Q1, Q3)** |  |  |  |  |  |  |  |  |
| HR (bpm) | 88.377 (76.353, 99.709) | 85.963 (75.681, 98.019) | 0.205 | 0.221 | 87.077 (74.333, 97.885) | 85.926 (75.385, 98.050) | 0.859 | 0.563 |
| RR (bpm) | 19.621 (17.281, 22.793) | 19.459 (16.944, 22.606) | 0.379 | 0.559 | 19.821 (17.062, 24.000) | 19.459 (16.944, 22.606) | 0.279 | 0.469 |
| SpO2 (%) | 97.347 (95.880, 98.592) | 97.120 (95.854, 98.288) | 0.433 | 0.245 | 97.309 (95.885, 98.585) | 97.120 (95.854, 98.288) | 0.687 | 0.304 |
| SBP (mmHg) | 113.623 (105.631, 126.564) | 111.925 (104.812, 125.899) | 0.453 | 0.660 | 110.034 (103.751, 119.875) | 111.925 (104.812, 125.899) | 0.185 | 0.257 |
| DBP (mmHg) | 61.329 (55.385, 67.970) | 60.288 (55.705, 69.207) | 0.694 | 0.733 | 60.383 (54.329, 65.733) | 60.288 (55.705, 69.207) | 0.423 | 0.282 |
| Temperature (℃) | 36.864 (36.548, 37.228) | 36.836 (36.457, 37.300) | 0.945 | 0.351 | 36.878 (36.565, 37.253) | 36.836 (36.457, 37.300) | 0.786 | 0.249 |
| **Laboratory tests, Median (Q1, Q3)** |  |  |  |  |  |  |  |  |
| cTnT (ng/mL) | 0.080 (0.040, 0.200) | 0.080 (0.042, 0.218) | 0.580 | 0.372 | 0.080 (0.040, 0.190) | 0.080 (0.042, 0.218) | 0.480 | 0.151 |
| WBC (10^3^/μL) | 14.200 (10.000, 20.175) | 13.750 (10.700, 20.275) | 0.931 | 0.731 | 15.200 (10.750, 22.600) | 13.750 (10.700, 20.275) | 0.292 | 0.313 |
| Hb (g/dL) | 11.400 (9.900, 13.100) | 10.450 (9.000, 11.900) | **<0.001** | 0.235 | 11.500 (9.900, 12.825) | 10.450 (9.000, 11.900) | 0.001 | 0.733 |
| PLT (10^3^/μL) | 206.000 (139.000, 280.000) | 237.500 (165.750, 320.750) | **0.020** | 0.250 | 198.000 (131.000, 282.250) | 237.500 (165.750, 320.750) | 0.016 | 0.216 |
| Cr (mg/dL) | 1.500 (1.000, 2.700) | 1.500 (0.900, 3.050) | 0.500 | 0.606 | 1.650 (1.100, 3.100) | 1.500 (0.900, 3.050) | 0.196 | 0.982 |
| BUN (mg/dL) | 30.000 (19.000, 49.750) | 31.000 (16.000, 50.750) | 0.299 | 0.543 | 32.500 (21.000, 56.250) | 31.000 (16.000, 50.750) | 0.183 | 0.275 |
| Lac (mmol/L) | 1.800 (0.000, 3.600) | 1.450 (0.150, 2.250) | **0.013** | 0.654 | 2.100 (1.100, 3.825) | 1.450 (0.150, 2.250) | 0.001 | 0.517 |
| **Comorbidity, N (%)** |  |  |  |  |  |  |  |  |
| Hypertension | 596 (42.755%) | 29 (30.851%) | 0.031 | 0.596 | 73 (44.512%) | 29 (30.851%) | 0.043 | 0.572 |
| DM | 473 (33.931%) | 23 (24.468%) | 0.077 | 0.123 | 48 (29.268%) | 23 (24.468%) | 0.493 | 0.203 |
| Liver disease | 257 (18.436%) | 17 (18.085%) | 1.000 | 0.729 | 37 (22.561%) | 17 (18.085%) | 0.489 | 0.932 |
| CKD | 368 (26.399%) | 22 (23.404%) | 0.605 | 0.170 | 39 (23.780%) | 22 (23.404%) | 1.000 | 0.208 |
| Malignancy | 241 (17.288%) | 11 (11.702%) | 0.209 | 0.684 | 29 (17.683%) | 11 (11.702%) | 0.272 | 0.144 |
| Dyslipidemia | 496 (35.581%) | 36 (38.298%) | 0.674 | 0.335 | 56 (34.146%) | 36 (38.298%) | 0.593 | 0.559 |
| **Treatments, N (%)** |  |  |  |  |  |  |  |  |
| IMV | 794 (56.958%) | 46 (48.936%) | 0.158 | 0.185 | 108 (65.854%) | 46 (48.936%) | 0.011 | 0.547 |
| CRRT | 41 (2.941%) | 0 (0.000%) | 0.107 | 0.355 | 9 (5.488%) | 0 (0.000%) | 0.028 | 0.320 |
| PICC | 475 (34.075%) | 43 (45.745%) | 0.029 | 0.325 | 67 (40.854%) | 43 (45.745%) | 0.526 | 0.462 |
| Vasopressor | 196 (14.060%) | 8 (8.511%) | 0.174 | 0.535 | 44 (26.829%) | 8 (8.511%) | 0.001 | 0.530 |
| **Medication, N (%)** |  |  |  |  |  |  |  |  |
| ACEIs | 235 (16.858%) | 14 (14.894%) | 0.726 | 0.551 | 24 (14.634%) | 14 (14.894%) | 1.000 | 0.810 |
| ARBs | 68 (4.878%) | 4 (4.255%) | 1.000 | 0.166 | 3 (1.829%) | 4 (4.255%) | 0.261 | 0.739 |
| beta blockers | 781 (56.026%) | 47 (50.000%) | 0.303 | 0.749 | 90 (54.878%) | 47 (50.000%) | 0.531 | 0.381 |
| **Critical assessment,**  **Median (Q1, Q3)** |  |  |  |  |  |  |  |  |
| SOFA | 7.000 (5.000, 10.000) | 6.000 (4.000, 9.750) | 0.055 | 0.346 | 8.000 (5.000, 13.000) | 6.000 (4.000, 9.750) | <0.001 | 0.348 |
| SAPS II | 42.000 (33.000, 52.000) | 37.000 (31.000, 48.250) | **0.011** | 0.106 | 46.000 (35.000, 57.000) | 37.000 (31.000, 48.250) | <0.001 | 0.233 |
| **Outcomes, N (%)** |  |  |  |  |  |  |  |  |
| 28-day mortality | 329 (23.601%) | 8 (8.511%) | 0.001 | 0.511 | 39 (23.780%) | 8 (8.511%) | 0.004 | 0.817 |
| 90-day mortality | 423 (30.344%) | 14 (14.894%) | 0.002 | 0.428 | 50 (30.488%) | 14 (14.894%) | 0.008 | 0.436 |
| 1-year mortality | 561 (40.244%) | 27 (28.723%) | 0.036 | 0.504 | 65 (39.634%) | 27 (28.723%) | 0.104 | 0.429 |

**Abbreviations:** PSM, propensity score matching; HR, heart rate; RR, respiratory rate; SBP, systolic blood pressure; DBP, diastolic blood pressure; SpO2, oxygen saturation of hemoglobin; Cr, creatinine; BUN, blood urea nitrogen; WBC, white blood cell; PLT, platelet; Hb, hemoglobin; Lac, lactate; CKD, chronic kidney disease; DM, diabetes mellitus; IMV, invasive mechanical ventilation; CRRT, continuous renal replacement therapy; PICC, peripherally inserted central catheter; SOFA, sequential organ failure assessment; SAPS II, simplified acute physiology score II‌; ACEIs, angiotensin converting enzyme inhibitors; ARBs, angiotensin II receptor blockers; SMD, standardized mean differences.
